# SUCROSE TRANSPORTER 5 supplies Arabidopsis embryos with biotin and affects triacylglycerol accumulation

**DOI:** 10.1111/tpj.12037

**Published:** 2012-12-31

**Authors:** Benjamin Pommerrenig, Jennifer Popko, Mareike Heilmann, Sylwia Schulmeister, Katharina Dietel, Bianca Schmitt, Ruth Stadler, Ivo Feussner, Norbert Sauer

**Affiliations:** 1 Molekulare Pflanzenphysiologie, Friedrich-Alexander Universität Erlangen-NürnbergStaudtstraße 5, D-91058, Erlangen, Germany; 2 Abteilung Biochemie der Pflanze, Albrecht von Haller Institut für Pflanzenwissenschaften, Georg August UniversitätJustus von Liebig Weg 11, D-37077, Göttingen, Germany

**Keywords:** biotin transport, embryo, fatty acid content, endosperm, sucrose transport, AtSUC5

## Abstract

The Arabidopsis SUC5 protein represents a classical sucrose/H^+^ symporter. Functional analyses previously revealed that SUC5 also transports biotin, an essential co-factor for fatty acid synthesis. However, evidence for a dual role in transport of the structurally unrelated compounds sucrose and biotin in plants was lacking. Here we show that SUC5 localizes to the plasma membrane, and that the *SUC5* gene is expressed in developing embryos, confirming the role of the SUC5 protein as substrate carrier across apoplastic barriers in seeds. We show that transport of biotin but not of sucrose across these barriers is impaired in *suc5* mutant embryos. In addition, we show that SUC5 is essential for the delivery of biotin into the embryo of biotin biosynthesis-defective mutants (*bio1* and *bio2*). We compared embryo and seedling development as well as triacylglycerol accumulation and fatty acid composition in seeds of single mutants (*suc5*, *bio1* or *bio2*), double mutants (*suc5 bio1* and *suc5 bio2*) and wild-type plants. Although *suc5* mutants were like the wild-type, *bio1* and *bio2* mutants showed developmental defects and reduced triacylglycerol contents. In *suc5 bio1* and *suc5 bio2* double mutants, developmental defects were severely increased and the triacylglycerol content was reduced to a greater extent in comparison to the single mutants. Supplementation with externally applied biotin helped to reduce symptoms in both single and double mutants, but the efficacy of supplementation was significantly lower in double than in single mutants, showing that transport of biotin into the embryo is lower in the absence of SUC5.

## Introduction

Biotin (vitamin B_7_ or vitamin H) is a prosthetic group in a small number of enzymes that catalyse essential carboxylation, decarboxylation and transcarboxylation reactions (Knowles, 1989; Nikolau *et al*., 2003; Smith *et al*., 2007; Ding *et al*., 2012). Its most prominent role is that of an essential co-factor for both the cytosolic and plastidic isoforms of acetyl CoA carboxylase, which both catalyse the first and rate-limiting step in fatty acid biosynthesis (Nikolau *et al*., 2003). Bacteria, plants, some fungi and a few animals are capable of synthesizing the biotin required for these reactions. All other organisms, including humans and baker's yeast (*Saccharomyces cerevisiae*), depend on uptake of biotin from their environment, and cDNAs of plasma membrane-localized biotin transporters have been cloned {*SODIUM-DEPENDENT MULTIVITAMIN TRANSPORTER* in human (*SDMT*; Prasad *et al*., 1998) and *VITAMIN H TRANSPORTER1* in yeast (*VHT1*; Stolz *et al*., 1999)}.

For identification of possible plant biotin transporters, a yeast mutant lacking the *VHT1* gene (Δ*vht1*) was complemented with an Arabidopsis cDNA library and screened for growth on medium containing low biotin concentrations (Ludwig *et al*., 2000). Surprisingly, this screening identified a sequence with high similarity to sucrose transporter cDNAs (e.g. *AtSUC1* and *AtSUC2* from Arabidopsis). Functional analyses of the encoded protein demonstrated that it was in fact a member of the Arabidopsis sucrose transporter family (named SUC5; At1g71890), with transport characteristics similar to those of previously published sucrose transporters (Ludwig *et al*., 2000). However, analyses with radiolabelled biotin confirmed that SUC5 also transports biotin (Ludwig *et al*., 2000), a molecule that has no structural similarity to sucrose.

Baud *et al*. (2005) showed *SUC5* expression mainly in the seed endosperm, in agreement with SUC5's potential role in biotin delivery into the seed, where large amounts of fatty acids are synthesized and stored as triacylglycerols (TAG). However, evidence for a role of SUC5 in catalysis of biotin transport *in planta* has so far not been provided. Analyses of three *suc5* mutants (*suc5.1*, *suc5.2* and *suc5.3*) studied from 4 to 22 days after fertilization (DAF) revealed that the fatty acid contents of mutant and wild-type were not or were hardly affected in 22 DAF seeds. However, 8 DAF *suc5* seeds showed a 20–45% transient decrease in their total fatty acid content. This time point (8 DAF) coincides with the onset of active fatty acid biosynthesis in developing Arabidopsis wild-type seeds (Baud 2002). Obviously, this transient phenotype may result from a reduction in SUC5-driven sucrose import (i.e. reduced availability of organic carbon for fatty acid biosynthesis), a reduction in SUC5-driven biotin supply (i.e. reduced availability of a co-factor required for fatty acid elongation), or a combination of both.

Here we describe use of physiological, genetic and biochemical approaches to elucidate the role of the SUC5 protein as a biotin transporter *in planta*, making use of T-DNA insertion mutants of *SUC5*. We isolated two allelic mutants disrupted in the *SUC5* gene (*suc5.4* and *suc5.5*), and used one (*suc5.4*) to measure uptake of the two putative substrates, biotin and sucrose, into isolated embryos. Analysis of substrate uptake into intact plants or embryos has been shown to be a suitable method to demonstrate *in planta* transport activity. Sherson *et al*. (2000) successfully analysed STP1-mediated transport of glucose by comparing uptake of {^14^C}-d-glucose into wild-type and *stp1* seedlings for up to 60 min. In another study, Matsuraka *et al*. (2000) incubated rice embryos for between 24 and 120 h with radiolabelled glucose, fructose and sucrose, and showed that uptake of {^14^C}-sucrose into embryos is driven by the action of the rice sucrose transporter OsSUT1. Our data also demonstrates that isolated Arabidopsis embryos are a suitable system for measuring active transport processes. In addition, we studied the physiological role of SUC5 by analysing developmental and biochemical properties of *suc5* transporter mutants in the background of Arabidopsis lines that are defective in biosynthesis of biotin, one of the putative substrates.

Arabidopsis plants with defects in biotin biosynthesis were first identified in analyses of embryo-lethal mutants. One mutant, *bio1.1*, was shown to be defective in synthesis of the biotin precursor 7,8-diaminopelargonic acid (Schneider *et al*., 1989; Muralla *et al*., 2008). The other mutant, *bio2.1*, is defective in conversion of dethiobiotin to biotin (Baldet and Ruffet, 1996; Patton *et al*., 1996, 1998; Weaver *et al*., 1996). The developmental arrest observed in homozygous (*bio1.1*/*bio1.1* or *bio2.1*/*bio2.1*) mutant embryos in the siliques of heterozygous (*BIO1*/*bio1.1* or *BIO2*/*bio2.1*) mother plants was rescued by watering the soil-grown heterozygotes with 0.5 mm biotin. This shows that the supplied biotin is taken up by the roots, translocated to the developing seeds, and imported into the endosperm and the developing embryo. This process requires transfer of biotin from the xylem into the sieve element/companion cell complexes of the phloem, which includes transport of biotin across several membranes. Exclusively passive diffusion of biotin across these various membranes seems unlikely.

We compared the development of embryos, seedlings and seeds in the various single mutants and *bio suc5* double mutants, and quantified the TAG content and the fatty acid composition in dry seeds of the various plants. Both *suc5* single mutants resembled wild-type plants, but the absence of SUC5 strongly enhanced all developmental and biochemical phenotypes observed in *bio1.1* and *bio2.1* mutant plants. In summary, our data show that SUC5 transfers biotin from the maternal tissue into the endosperm and embryos of developing Arabidopsis seeds, and that this activity is essential under conditions of biotin limitation.

## Results

### Analyses of p*SUC5*/*sGFP* and p*SUC5*/*tmGFP9* plants

**Figure 1:**
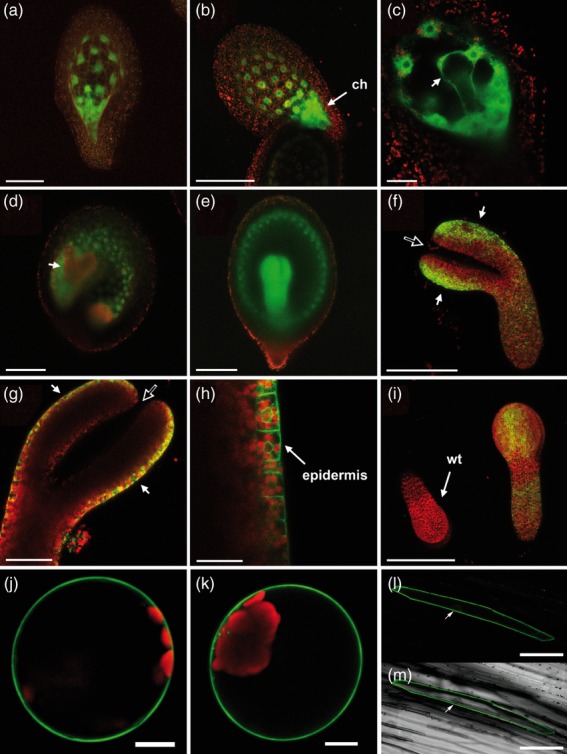
Analysis of p*SUC5*/*sGFP* and p*SUC5*/*tmGFP9* plants and subcellular localization of SUC5. (a–i) Confocal images of developing seeds (a–e) and isolated embryos (f–i) from p*SUC5*/*sGFP* plants (a, d, e) or p*SUC5*/*tmGFP9* (b, c, f–i) plants. (a) Young seed with syncytial endosperm and no detectable embryo, showing GFP fluorescence in the endosperm nuclei (maximum projection). (b) Young seed with syncytial endosperm showing strong GFP fluorescence in the chalazal region (ch) (maximum projection). (c) Developing seed showing GFP fluorescence (optical section) in the ER of the endosperm but not in the globular embryo (arrow). (d) Slightly older seed showing GFP fluorescence in the endosperm nuclei (maximal projection) but not in the heart-stage embryo (arrow). (e) Developing seed showing GFP fluorescence in the early torpedo-stage embryo and the endosperm. (f, g) Isolated walking stick-stage embryo {maximum projection (f), optical section (g)} showing highest GFP fluorescence at the anatomical underside of the developing cotyledons (white arrows), no GFP fluorescence on the upper side (black arrow), and little fluorescence in the hypocotyl. (h) Higher magnification (optical section) of a section through the underside of a forming cotyledon {same embryo as in (g)}, showing GFP fluorescence in the epidermis only. (i) Comparison of fluorescence in an isolated wild-type embryo {slightly older (mid-torpedo stage) than the embryo shown in (e)} and a p*SUC5*/*tmGFP9* embryo {similar stage as in (f) and (g)}. (j–m) Transient expression of GFP–SUC5 (j, l) and SUC5–GFP (k, m) fusion proteins in Arabidopsis protoplasts (j, k) and leek epidermis cells (l, m). Arrowheads in (l) and (m) indicate the nucleus. The red colour results from autofluorescence of chlorophyll. Scale bars = 50 μm (a), 100 μm (b, d, e, g, l), 25 μm (c), 200 μm (f, i) and 10 μm in (h, j, k).

We generated two p*SUC5*/reporter lines under the control of a 2030 bp *SUC5* promoter. These lines expressed the open reading frames of soluble and freely mobile green fluorescent protein (sGFP) or a non-mobile version of GFP (tmGFP9) that is membrane-attached via N-terminal transmembrane helices (Stadler *et al*., 2005a). Analyses of these plants by confocal microscopy confirmed the previously reported *SUC5* expression in the endosperm (Figure 1a,b) (Baud *et al*., 2005), and accumulation of GFP at the chalazal end of the endosperm (Figure 1a,b). No expression of GFP was observed in globular (Figure 1c) or heart-stage embryos (Figure 1d). Unexpectedly, we observed p*SUC5* activity for both constructs during the later stages of embryo development (Figure 1e–i). This *SUC5* expression in the embryo was not observed in earlier analyses performed using standard fluorescence microscopy and a shorter 1500 bp promoter fragment (Baud *et al*., 2005).

Optical sections through p*SUC5*/*tmGFP9* embryos confined p*SUC5* activity specifically on the epidermis of the outer surface of the cotyledons (Figure 1g,h). In previous analyses (Stadler *et al*., 2005a), it was shown that sGFP synthesized in the embryo epidermis from Arabidopsis *GLABRA2* promoter (p*GL2*)/*sGFP* constructs moves symplasmically into all other cells of the developing embryo due to the presence of large plasmodesmata. This explains the homogenous fluorescence seen in early torpedo-stage embryos expressing *sGFP* from p*SUC5* (Figure 1e). Embryos from wild-type seeds showed no fluorescence at any developmental stage (Figure 1i).

In addition, we analysed the subcellular localization of the SUC5 protein by transiently transforming Arabidopsis protoplasts with N- and C-terminal GFP fusion constructs of SUC5. Both fusion proteins localized to the plasma membrane (Figure 1j,k). We verified this localization in particle-bombarded leek (*Allium ampeloprasum*) epidermis cells (Figure 1l,m).

### Identification of two allelic mutant lines defective in *SUC5*

**Figure 2:**
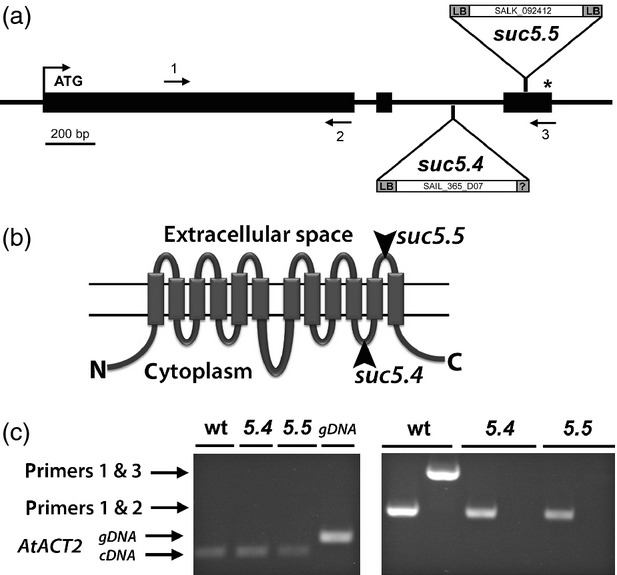
Characterization of the *suc5.4* and *suc5.5* mutant alleles in Col-0. (a) Schematic of the *SUC5* gene showing three exons (thick lines) and the T-DNA insertions in the *suc5.4* and *suc5.5* mutants. ATG indicates the start codon and the asterisk indicates the stop codon. Arrows show the positions and directions of primers used in (c). (b) Schematic of the SUC5 protein. The 12 predicted transmembrane helices are shown. Arrowheads indicate the sites where the protein is disrupted by the T-DNA insertions. (c) Left: cDNA and genomic fragments obtained with PCR using *ACTIN2*-specific primers on cDNA of wild-type, *suc5.4* and *suc5.5* or wild-type genomic DNA (gDNA). Right: cDNA fragments obtained with combinations of primers 1, 2 and 3 (amplifying various regions of *SUC5*) on cDNA from wild-type, *suc5.4* and *suc5.5*. Bands indicating the presence of an intact *SUC5* mRNA were not amplified in the mutants, but were obtained in the wild-type.

The *suc5.1*, *suc5.2* and *suc5.3* mutants described by Baud *et al*. (2005) were generated in the Wassilewskija background. As the *bio1.1* and *bio2.1* mutants were in ecotype Columbia (Col-0), we characterized two *suc5* mutants (SAIL_365_D07, *suc5.4*; SALK_092412, *suc5.5*; http://signal.salk.edu/cgi-bin/tdnaexpress) in the same ecotype. These mutants carry insertions after nucleotide 250 of the 2nd intron (*suc5.4*) or between nucleotides 98 and 105 of the 3rd exon (*suc5.5*, six nucleotides deleted), with *suc5.5* having a tandem insertion with the two right borders facing each other (Figure 2a). For the *suc5.4* allele, only the orientation of the left border was determined. Comparative RT-PCR using RNA isolated from flower tissue (Figure 2c) demonstrated that both lines produced mRNA from the first exon, but failed to produce the complete *SUC5* mRNA. Therefore, both mutants produce truncated SUC5 proteins, with the T-DNA insertion in *suc5.4* resulting in a truncated protein lacking the last two transmembrane helices, and the T-DNA insertion in *suc5.5* resulting in a truncated protein lacking the last transmembrane helix. Studies on the structurally related HUP1 hexose transporter of *Chlorella* demonstrated that the C-terminal region and the 11th transmembrane domain in particular are essential for transport function, and it is therefore unlikely that the truncated SUC5 proteins analysed here retain any transport activity (Caspari *et al*., 1994; Will *et al*., 1998).

### Uptake of biotin and sucrose by wild-type and *suc5* embryos

**Figure 3:**
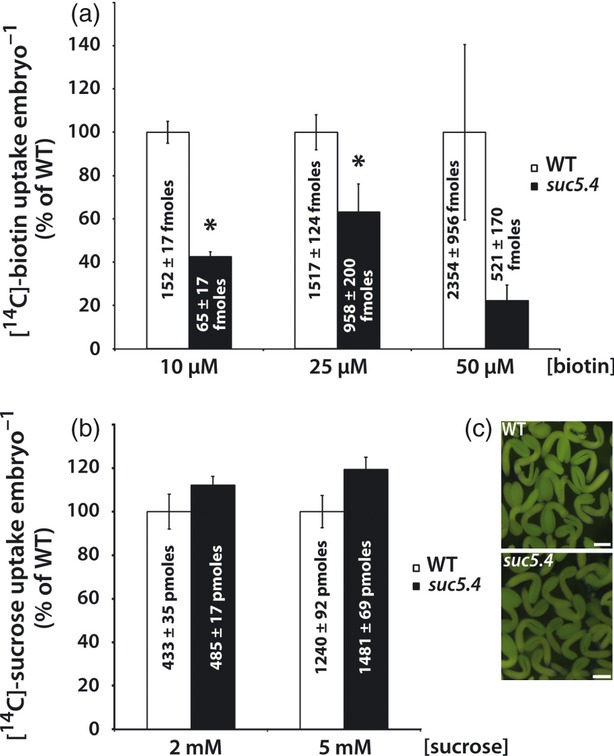
Uptake of biotin and sucrose by wild-type and *suc5.4* embryos. (a) Uptake of {^14^C}-biotin (three concentrations) into wild-type and *suc5.4* embryos at 8 DAF. The amount of biotin taken up per embryo after 6 h is indicated on each bar. (b) Uptake of {^14^C}-sucrose (two concentrations) into wild-type and *suc5.4* embryos at 8 DAF. The amount of sucrose taken up per embryo after 90 min is indicated on each bar. Values in (a) and (b) are means ± standard errors from three independent measurements at the indicated concentration. (c) Isolated wild-type and *suc5.4* embryos at 8 DAF used for uptake measurements with radiolabelled biotin or sucrose. Scale bars = 250 μm.

SUC5-mediated transport of biotin was demonstrated by heterologous expression of *SUC5* in a yeast strain lacking VHT1, the endogenous transporter for biotin (Ludwig *et al*., 2000). To confirm the biotin transport activity of SUC5 *in planta*, we compared the uptake of radiolabelled biotin and sucrose into isolated embryos (8 DAF) from *suc5.4* single mutants and wild-type (Figure 3). The embryo is separated from the maternal tissue by the seed apoplasmic space, and therefore import into the embryo may only be driven by active transport processes. These uptake measurements revealed that the uptake rate of biotin into isolated wild-type embryos was concentration-dependent, and that the uptake of biotin into *suc5.4* embryos was significantly lower (for 10 and 25 μm biotin; *P* < 0.05, Student's *t*-test) than that into wild-type embryos (Figure 3a). In contrast to biotin, uptake of sucrose was unaltered in wild-type and *suc5.4* (Figure 3b). No phenotypic differences were observed between the *suc5.4* and wild-type embryos (Figure 3c).

### Comparative analyses of embryo and seed development

The originally described *bio1.1* and *bio2.1* mutations (Schneider *et al*., 1989; Patton *et al*., 1998) were induced by chemical mutagenesis. Homozygous *suc5.4* or *suc5.5* plants were crossed with these *bio1.1* or *bio2.1* mutants, and double homozygosity was determined by PCR (for the T-DNA insertions in *suc5.4* and *suc5.5*) and by confirming the growth defect on biotin-free medium (for *bio1.1* and *bio2.1*) in the subsequent generations.

On soil, these double homozygotes developed normal rosettes, flowered and produced fertile seeds when watered with 1 mm biotin. Without supplemented biotin, the homozygous *bio1.1* and *bio2.1* plants and all double homozygotes germinated poorly, and developed into tiny plants that turned pale and eventually died (Figure S1). However, when the seeds were first germinated on biotin-containing (1 mm) agar medium for approximately 10 days and then transferred to soil, normal-looking plants developed that flowered even without further biotin supplementation (Figure S2).

To compare the development of seeds and embryos in wild-type plants, *suc5.4*, *suc5.5*, *bio1.1* and *bio2.1* single mutants and *bio* *suc5* double mutants, seeds of all lines were germinated on agar medium containing 1 mm biotin. After 12 days, all seedlings were an identical size and were transferred to soil (Figure S2). These seedlings were then watered either without additional biotin, with 0.1 mm supplemental biotin, or with 1 mm supplemental biotin.

**Figure 4:**
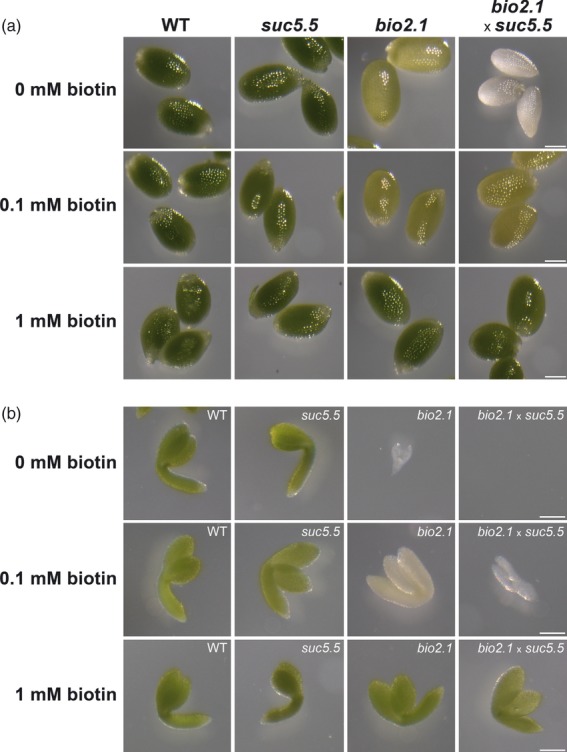
Development of seeds and embryos in siliques of wild-type plants and various single and double mutants. After 12 days on agar medium with 1 mm biotin, plants were transferred to soil and watered with the indicated supplementation of biotin. Scale bars = 200 μm in. (a) Developing seeds isolated from siliques of comparable developmental stages. (b) Embryos isolated from the developing seed batches analysed in (a).

We harvested siliques at the same developmental stage from these plants, and analysed seed and embryo development (Figure 4a,b). Whereas developing wild-type and *suc5* seeds and embryos looked normal under all growth conditions, *bio2.1* seeds and embryos showed a biotin-dependent phenotype. Without biotin supplementation, the *bio2.1* seeds were yellowish and pale, and embryos isolated from these seeds showed strongly retarded development (0 mm biotin, Figure 4a,b). This defect was partly rescued in plants supplemented with 0.1 mm biotin. Embryos from these plants were the same size as wild-type embryos; however, they were unable to synthesize chlorophyll and had a faint yellowish colour. Embryos from *bio2.1* plants supplemented with 1 mm biotin were able to synthesize chlorophyll and looked essentially like wild-type embryos.

A significantly stronger phenotype was observed in developing seeds and embryos of *bio2.1* *suc5.5* double mutants that were not supplemented with biotin (0 mm biotin, Figure 4a,b). Seeds from these plants were white and smaller than seeds of *bio2.1* single mutants, indicating a stronger biotin deficiency. Without biotin supplementation, no embryos were detected in these seeds, and even embryos from plants supplemented with 0.1 mm biotin showed a strong developmental phenotype. Only in seeds from plants that were supplemented with 1 mm biotin were wild-type-like embryos formed.

**Figure 5:**
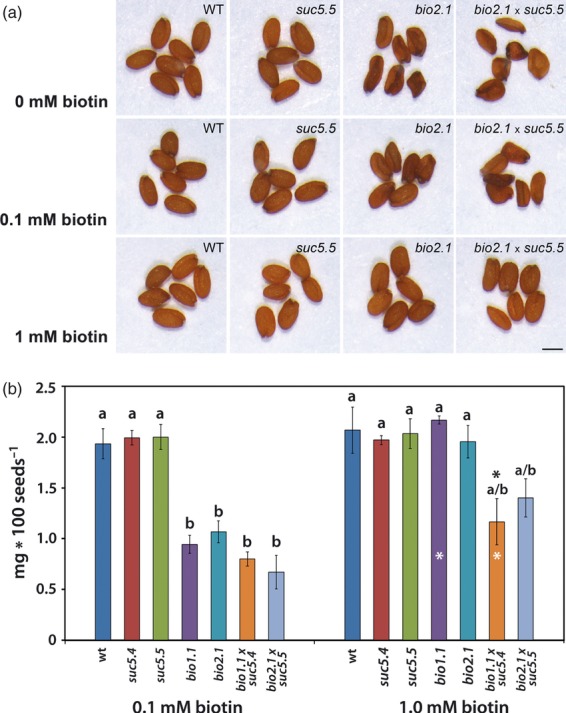
Phenotypes and 100-seed weight of dry seeds from wild-type plants, and single and double mutants supplemented with various biotin concentrations. (a) Dry seeds from the indicated plant lines. Seeds of wild-type and *suc5.5* plants are shaped normally under all growth conditions. Seeds of *bio2.1* plants are wrinkled and seeds of *bio2.1 suc5.5* double mutants have the appearance of ‘empty bags’ when their parent plants are not supplemented with biotin. Supplementation with 0.1 mm biotin complemented this defect partly (*bio2.1* seeds are almost normal looking; *bio2.1* *suc5.5* seeds are still wrinkled). Seeds looked normal when the parent plants were watered with 1 mm biotin. Scale bars = 500 μm. (b) Weight of dry seeds in mg per 100 seeds for wild-type and the indicated genotypes supplemented with either 0.1 or 1.0 mm biotin. Bars labelled with different letters represent 100-seed weights that differ significantly from each other (*P* ≤ 0.005, Student's *t*-test). The difference between *bio1* and *bio1.1 suc5.4* at 1 mm (white asterisks) is slightly significant (*P* ≤ 0.05, Student's *t*-test, black asterisk above bar).

We finally compared the morphology (Figure 5a) and the weight (Figure 5b) of dry seeds from homozygous single and double mutants, and from wild-type plants. As expected, wild-type and *suc5.5* mutant seeds looked normal under all growth conditions. In contrast, the seeds of *bio2.1* and *bio2.1* *suc5.5* plants had a slightly (*bio2.1*) or strongly wrinkled appearance (*bio2.1* *suc5.5*) when the plants had not been supplied with biotin. Supplementation of 0.1 mm biotin complemented these phenotypes to various extents. Whereas the *bio2.1* seeds looked almost normal, all of the *bio2.1* *suc5.5* seeds still had a wrinkled appearance. Watering of the parent plants with 1 mm biotin completely (*bio2.1*) or almost completely (*bio2.1* *suc5.5*) reversed this phenotype. Similar results were obtained for developing seeds and embryos and for dry seeds from *bio1.1* and *bio1.1* *suc5.4* plants.

Because only very few seeds were obtained from *bio suc5* double mutants that were watered without any biotin supplementation, we compared the seed weight only from plants supplemented with 0.1 and 1 mm biotin. The 100-seed weight from wild-type, *suc5.4* and *suc5.5* mutants was unaltered under both supplementation conditions (0.1 and 1 mm biotin, Figure 5b), but that of *bio1.1*, *bio2.1*, *bio1.1 suc5.4* and *bio2.1 suc5.5* mutants was significantly lower (*P* ≤ 0.005, Student's *t*-test) than in wild-type or *suc5* single mutants under both conditions. The seed weight of *bio1.1 suc5.4* and *bio2.1 suc5.5* was also lower than that of the *bio1.1* and *bio2.1* single mutants, but this was not significant for 0.1 mm biotin. For 1 mm biotin, a significant difference was observed for *bio1.1* versus *bio1.1 suc5.4* (indicated by an asterisk, *P* ≤ 0.05, Student's *t*-test). The differences in seed weight reflect the observed morphological differences in these seeds.

### Phenotypic comparison of seedlings

**Figure 6:**
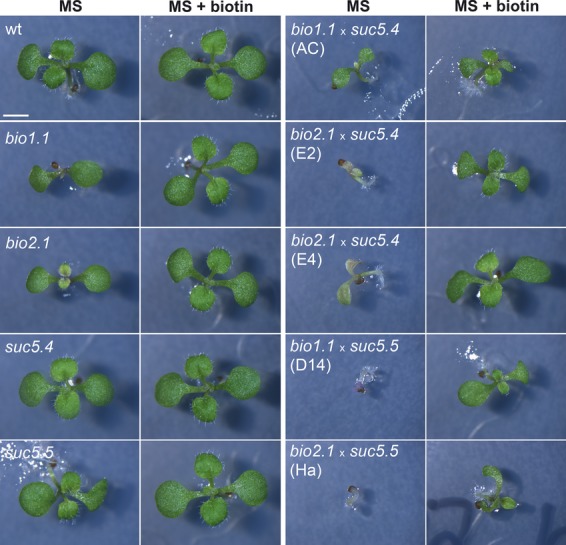
Comparative analysis of 10-day-old seedlings from wild-type plants and homozygous single or double mutants on MS medium or MS medium supplemented with biotin. Parent plants of all seeds with a *bio* mutation were watered with 1 mm biotin. The names of various double mutant lines are given in parentheses. Scale bar = 2 mm.

Homozygous single mutant seedlings (*bio1.1*, *bio2.1*, *suc5.4* and *suc5.5*) developed normally on high-biotin medium and were indistinguishable from wild-type plants (Figure 6, MS + biotin). However, similar analyses on biotin-free medium (Figure 6, MS) confirmed the previously described defects for *bio1.1* and *bio2.1* seedlings (Schneider *et al*., 1989; Patton *et al*., 1998). Germinable homozygous *bio1.1* and *bio2.1* seeds were obtained exclusively from biotin-watered plants. After germination, the seedlings started to form normally sized, green cotyledons. However, the first pair of rosette leaves developed poorly, and was tiny and yellowish (Figure 6), and eventually these plants died before developing inflorescences. In contrast, *suc5.4* and *suc5.5* (parent plants not watered with biotin) showed no phenotypic difference from wild-type seedlings on biotin-free medium.

When the same analyses were performed using seeds of *bio1.1 suc5.4*, *bio1.1 suc5.5*, *bio2.1 suc5.4* and *bio2.1 suc5.5* double homozygotes (all parent plants watered with biotin; Figure 6, right panel), we observed a high percentage of seedlings with severely aberrant cotyledon phenotypes on biotin-free medium (Figure 6, MS). The cotyledons of almost all seedlings were smaller than those of wild-type seedlings, and these cotyledons were partially or completely white in a high percentage of seedlings. Interestingly, even these strong phenotypes were rescued by 1 mm biotin in the growth medium (Figure 6, MS + biotin). However, the development of these rescued double mutant seedlings was delayed compared to rescued *bio1.1* or *bio2.1* seedlings.

**Figure 7:**
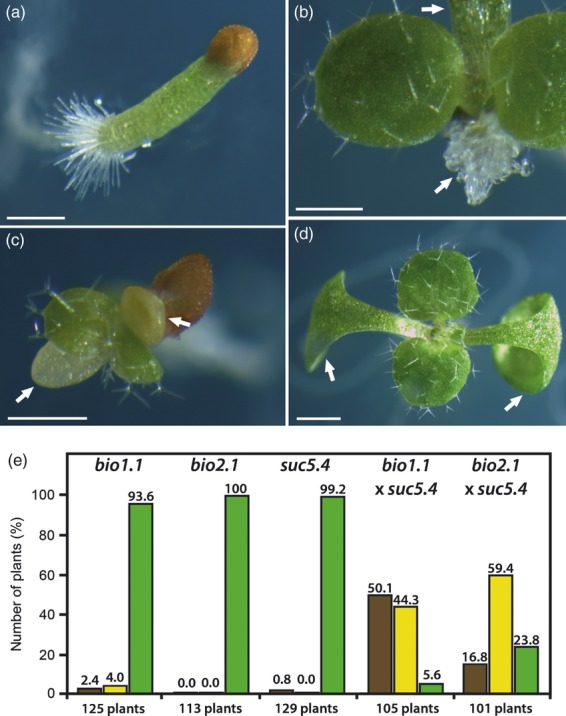
Phenotypes of 12-day-old double homozygous *bio1* *suc5* and *bio2 suc5* seedlings. (a) *bio1.1 suc5.4* seedling with no visible cotyledons. (b) *bio2.1 suc5.5* seedling with a green cotyledon (upper arrow) and a white, callus-like cotyledon (lower arrow). (c) *bio1.1 suc5.4* seedling with small yellowish cotyledons (arrows). (d) Seedling of a wild-type plant with normal cotyledons (arrows) photographed at the same age as the mutants in (a–c). (e) Quantification of phenotypes. Green bars show the percentage of seedlings forming green, normally shaped cotyledons, yellow bars show the percentage of seedlings with severe cotyledon phenotypes {as in (a–c) or similar}, brown bars show the percentage of seeds that were not yet germinated at the time of analysis. The total number of plants analysed is shown below the bars. Scale bars = 500 μm (a–c) and 1 mm (d).

A more detailed and quantitative analysis of these double mutant phenotypes is shown in Figure 7. The cotyledons of many seedlings failed to expand and were too small to lift the seed coat (Figure 7a). However, even when the expanding cotyledons lifted the seed coat, they stayed tiny and failed to turn green (Figure 7c). Other seedlings had mis-shapen, only partially green cotyledons (Figure 7b), and the few seedlings with normally shaped cotyledons were smaller than wild-type seedlings (Figure 7d). A significant number of seeds did not germinate at all or only after prolonged incubation. A quantification of the observed phenotypes (Figure 7e) demonstrated that the percentage of mis-shapen seedlings or non-germinating and late-germinating seeds was negligible in the various homozygous single mutants, but very high in the double mutants.

### Analyses of TAG content and fatty acid composition in seeds

**Figure 8:**
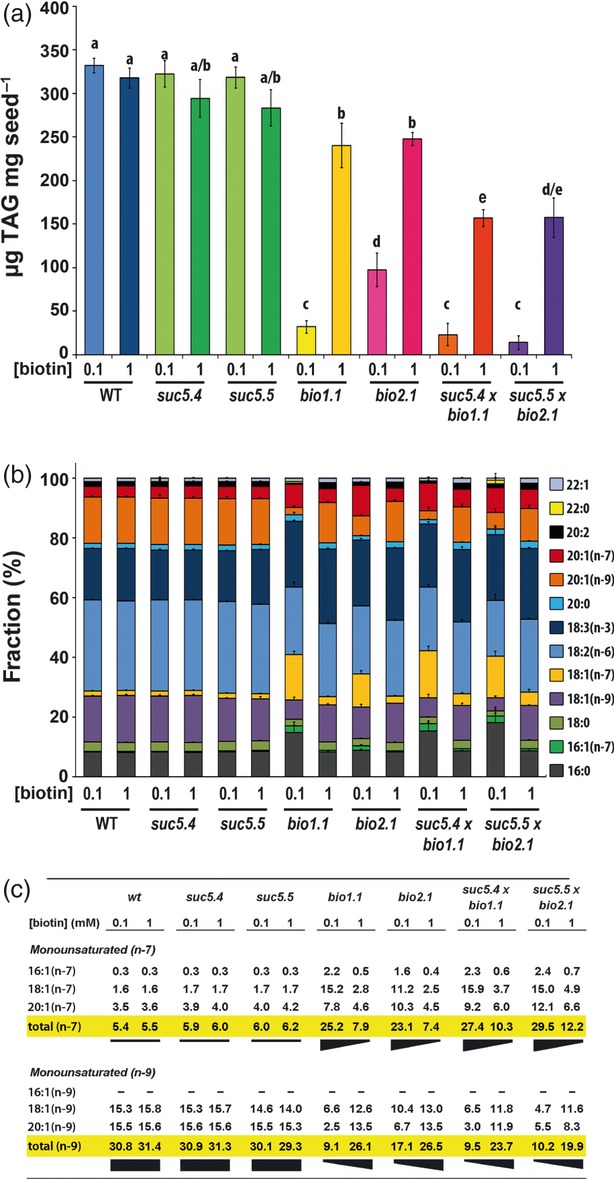
TAG content of dry seeds, and fatty acid composition of these TAGs. (a) TAG content in seeds from wild-type plants, and from single and double mutants that were supplemented with the indicated biotin concentrations. Bars labelled with different letters (a–e) represent TAG contents that differ significantly from each other (*P* ≤ 0.005, Student's *t*-test). TAG contents labelled with two letters (a/b and d/e) do not differ significantly from either a and b or d and e. (b) Fatty acid composition in the TAGs shown in (a). (c) Mol% values of (*n*-7) and (*n*-9) fatty acids extracted from the data shown in (b). In wild-type, *suc5.4* and *suc5.5* seeds, the content of (*n*-9) fatty acids is approximately six times higher than that of (*n*-7) fatty acids, and these values are not affected by added biotin. However, in seeds of *bio1.1* and *bio2.1* plants, and even more so in seeds of *bio1.1 suc5.4* and *bio2.1* *suc5.5* plants, the mol% of (*n*-7) and (*n*-9) fatty acids are increased or decreased, respectively, and these changes are reversed towards wild-type levels by higher biotin concentrations. Thick bars, thin bars or triangles indicate high, low, increasing or decreasing mol% values, respectively.

As biotin-dependent enzymes are involved in central steps of fatty acid biosynthesis (Ohlrogge and Jaworski, 1997), biotin limitation not only affects seed and seedling morphology, but also reduces the capacity to synthesize TAG. If SUC5 acts as biotin transporter, an additional *suc5* mutation should further reduce these TAG levels. The seed yield from double mutants that were not supplemented with biotin was not high enough to perform TAG analyses. Therefore, we analysed seeds from wild-type plants, single and double mutants that were supplemented with 0.1 or 1 mm biotin (Figure 8a). For 0.1 mm biotin, the total TAG content of seeds from *bio1.1* and *bio2.1* single mutants was reduced to 10% (*bio1.1*) and 30% (*bio2.1*) of wild-type levels. For 1 mm biotin, the TAG content of the single mutants was still significantly lower than that of similarly supplemented wild-type plants (75% for *bio1.1* and 78% for *bio2.1*). This reduction in TAG content was even greater in seeds from *bio* *suc5* double mutants. The TAG content of *bio1.1 suc5.4* seeds was only 6% (0.1 mm biotin) and 46% (1 mm biotin) of wild-type levels, and that of *bio2.1 suc5.5* seeds was only 4% (0.1 mm biotin) and 49% (1 mm biotin) of wild-type levels. This stronger reduction also resulted in significant lower TAG contents between *bio* single and *bio suc5* double mutant seeds (*P* ≤ 0.005, Student's *t*-test). Only the difference between *bio1.1* and *bio1.1 suc5.4* supplemented with 0.1 mm biotin was not significant. Seeds from *suc5.4* and *suc5.5* single mutant plants supplemented with 0.1 or 1 mm biotin had TAG levels that were similar to the wild-type (Figure 8a).

Analyses of the fatty acid composition in the various TAG samples revealed that the reduced TAG contents in seeds of *bio1.1* and *bio2.1* plants (0.1 mm biotin) were paralleled by altered fatty acid compositions. Five- to sixfold higher contents of mono-unsaturated (*n*-7) fatty acids {16:1(*n*-7), 18:1(*n*-7) and 20:1(*n*-7)} and two- to threefold lower contents of mono-unsaturated (*n*-9) fatty acids {18:1(*n*-9) and 20:1(*n*-9); Figure 8b,c} were detected. Moreover, these seeds showed a higher percentage of the short-chain fatty acids 16:0 and 16:1(*n*-7).

All of these alterations were more pronounced (up to twofold) in *bio1.1 suc5.4* and *bio2.1 suc5.5* seeds (Figure 8b,c). The percentage of the short-chain fatty acids 16:0 and 16:1(*n*-7) increased to over 20 mol% in *suc5.5 bio2.1* in 0.1 mm biotin, in comparison with 8.5 mol% in wild-type, *suc5.5* or *bio2.1* in 0.1 mm biotin.

The fatty acid composition in seeds from *bio1.1* or *bio2.1* plants supplemented with 1 mm biotin were comparable to those in *suc5.5* or wild-type seeds, but we did not observe complete restoration of the wild-type distribution in seeds of *bio suc5* plants (Figure 8c). The seeds of *suc5* or wild-type plants showed the same fatty acid composition under both conditions (Figure 8c).

## Discussion

The studies described here address the question of whether or not the Arabidopsis SUC5 protein acts as a biotin transporter *in planta*.

When we started our analyses, it was known that SUC5 transports both sucrose and biotin in yeast cells (Ludwig *et al*., 2000), that the *SUC5* gene is expressed in the endosperm, and that *suc5* mutants accumulate wild-type TAG levels in dry seeds (Baud *et al*., 2005).

Here we show that the SUC5 protein localizes to the plasma membrane (Figure 1j–m), confirming the predicted localization for a sucrose transporter of the SUT-1 clade (Reinders 2012; Wolfenstetter *et al*., 2012). Moreover, *SUC5* is also expressed in the epidermis of torpedo-stage or older embryos (Figure 1e–i), demonstrating that SUC5 is involved in transport of its substrate(s) across the plasma membrane of embryo epidermis cells. Our results also demonstrate that SUC5 is important for transport of biotin across these boundaries, and provide evidence that biotin transport by SUC proteins is physiologically relevant *in planta*.

### SUC5 is responsible for biotin transport *in planta*

In agreement with our localization data (Figure 1), *suc5* single mutants displayed reduced uptake of biotin but not sucrose into 8 DAF embryos in comparison with wild-type (Figure 3a,b), suggesting that either the role of SUC5 for the transport of sucrose into the embryo is negligible or the lack of sucrose transport activity is compensated for by the action of other SUCs. Of the nine members of the SUC transporter family in Arabidopsis, localization in the developing seed has been shown for SUC3 (embryo radicle; Meyer *et al*., 2004), SUC8 (whole seed; Sauer, 2007) and SUC9 (embryo cotyledons plus radicle; Sivitz *et al*., 2007). SUC9 in particular appears to be a good candidate for assuming a primary role in sucrose loading into the embryo, because it resembles SUC5 in terms of its transport kinetics for sucrose but has a tenfold lower *K*_m_ for sucrose (<0.1 mm for SUC9 versus 1 mm for SUC5; Ludwig *et al*., 2000; Sivitz *et al*., 2007). However, detailed analyses of *AtSUC9* mutant embryos have not been performed, and nor has biotin transport activity for AtSUC9 been investigated yet.

However, the measured difference in biotin uptake suggests that, although the spatio-temporal expression patterns of the corresponding genes encoding the above mentioned SUCs overlap with that of *SUC5*, these transporters cannot fully compensate for the missing biotin transport activity of SUC5. The nonetheless measurable accumulation of radiolabelled biotin in *suc5* mutant embryos may result from a lower transport affinity for biotin of these other SUCs (as shown for PmSUC2; Ludwig *et al*., 2000).

Apart from the reduced uptake of biotin into *suc5.4* embryos (Figure 3), our comparisons of wild-type plants and *suc5* mutants revealed no differences in embryo and seedling development (Figures 4-6), no significant decrease in TAG accumulation and no alteration in fatty acid compositions (Figure 8).

*bio1.1* and *bio2.1* single mutants had severe phenotypes, such as impaired seedling development (Figure 6), altered seed morphology and weight (Figures 4 and 5), reduced TAG content (Figure 8a), and altered fatty acid composition in dry seeds (Figure 8b,c). Most importantly, mutations in the *SUC5* gene in the *bio1* or *bio2* mutant backgrounds led to a significant increase of all these defects. *bio1 suc5* and *bio2 suc5* double mutants that were not supplemented with biotin for one generation produced almost no seeds, and the few seeds obtained showed drastically impaired germination (Figure 7b). In fact, the *bio1* *suc5* and *bio2 suc5* seeds (Figure 5) looked empty, had a wrinkled appearance, and resembled seeds of the low seed-oil mutant *wrinkled1* (*wri1*; Focks and Benning, 1998), which has a defect in a transcription factor (WRI1) that is involved in control of metabolism, particularly fatty acid synthesis, in developing seeds (Cernac *et al*., 2006; Baud *et al*., 2007). Seedlings that germinated from these *bio1 suc5* and *bio2 suc5* seeds exhibited strong deformations of their cotyledons, the main site of TAG synthesis and storage in the developing embryo (Figure 7a–c). The observed differences in cotyledon deformation may reflect variations in the biotin content of individual seeds caused by an unequal supply of biotin under conditions of biotin limitation. As in the *bio1* and *bio2* single mutants, supplementation with externally applied biotin established the wild-type phenotype in the *bio1 suc5* and *bio2 suc5.5* double mutants. However, full restoration of wild-type parameters, especially seed weight (Figure 5b), TAG content and TAG composition (Figure 8a–c) was not observed. This most likely reflects the lack of biotin transport activity in the embryo mediated by SUC5, and suggests that possibly even higher biotin concentrations are required for non-SUC5 mediated transport of biotin into the embryo.

When the *bio* or *bio1 suc5* and *bio2 suc5* mutants were supplemented with biotin, their otherwise embryo-lethal defects were rescued. Following uptake of biotin by the roots, and its translocation via the xylem, it is eventually unloaded at the integument, where both phloem and xylem vessels terminate (Stadler *et al*., 2005a). Transfer of biotin from the xylem into the phloem involves the apoplast and is probably carrier-mediated, but it is unclear which carriers load biotin into sieve element/companion cell complexes. Complementation studies in yeast suggest an involvement of SUT1-type sucrose transporters, as shown for PmSUC2 (Ludwig *et al*., 2000). Export from the maternal tissues into the seed apoplasmic space is probably also carrier-mediated. Recently, a group of carriers has been described that catalyses the release of sucrose into the apoplast (SWEET proteins, Chen *et al*., 2012). Whether these sucrose efflux transporters also accept biotin as a second substrate is unknown. However, our data suggest that import of biotin into the endosperm and the embryo epidermis is catalysed by the SUC5 protein. At what rate this import is catalysed is unknown, because the actual local biotin concentration at the integument and the outside of the embryo epidermis vary during embryo development. However, in biotin transporter-deficient yeast cells complemented with SUC5, biotin import was measured over a concentration range between 5 μm and 2 mm biotin, and such complemented yeast cells grow on medium with biotin concentrations as low as 10 nm (Ludwig *et al*., 2000).

### Sufficiently high biotin levels are essential for fatty acid and TAG biosynthesis

In comparative analyses of embryo and endosperm fatty acid contents, Penfield *et al*. (2004) found that the endosperm contains proportionally higher levels (approximately 20%) of (*n*-7) long-chain fatty acids {16:1(*n*-7), 18:1(*n*-7) and 20:1(*n*-7)} than the embryo (2%). In contrast, 20:1(*n*-9) levels were shown to be proportionally higher in the embryo. The changes in the fatty acid compositions in *bio* versus *bio suc5* seeds (Figure 8c) may therefore indicate that the reduced TAG levels (Figure 8a) in these seeds coincide with an increase in the endosperm-to-embryo ratio. The observed patterns of seed and embryo development shown in Figure 4 support this interpretation.

The embryo represents a strong sink for biotin, and our data suggest that fatty acid biosynthesis and acetyl CoA carboxylase activity are affected by changes in the availability of biotin. Reduced or absent SUC5 activity eventually leads to a lack of biotin in the embryo. This lack may cause the previously described transient decrease in fatty acid content in *suc5* mutants at 8 DAF (the onset of fatty acid biosynthesis) (Baud *et al*., 2005). This lack of biotin is rapidly overcome by the biotin synthesis in developing wild-type seeds, but becomes increasingly important under conditions of biotin limitation (e.g. in *bio1* or *bio2* mutants). In addition to biotin biosynthesis, biotin supply from adjacent tissues is an alternative mechanism to adjust cellular biotin concentrations.

Our data suggest that the concentration of biotin is adjusted to the specific needs of an organ under various developmental conditions, and that SUC5 participates in this adjustment. An essential role for SUC5 in the supply of sucrose is rather unlikely, as, in *suc5* embryos, neither the sucrose import is altered (Figure 3b) nor is the total fatty acid or TAG composition in dry seeds affected (Figure 8c) (Baud *et al*., 2005). The expression kinetics of *SUC5* during seed development reported by Baud *et al*. (2005) support a scenario in which the primary role of SUC5 is biotin transport. *SUC5* shows peak expression at 7 DAF, which coincides with the onset of fatty acid synthesis. Shortly after, *SUC5* expression decreases to almost zero and stays low during later seed development and ripening. Possibly, a short time frame, during which transport of the essential co-factor biotin across the embryo epidermis is catalysed by SUC5, is sufficient for proper fatty acid synthesis. Other genes encoding SUCs that are localized in the embryo, namely SUC8 and SUC9, are also active at later developmental stages (Sauer, 2007; Sivitz *et al*., 2007), and may provide the embryo with the carbon skeletons required for fatty acid synthesis. It remains to be shown whether or not biotin transport is a physiologically important property of other plant sucrose transporters also.

## EXPERIMENTAL PROCEDURES

### Plant materials and growth conditions

*Arabidopsis thaliana* Col-0 and mutant plants were grown in a growth chamber on potting soil under a 16 h light/8 h dark regimen at 22°C and 60% relative humidity, and watered with the indicated biotin concentrations. *bio1.1* (NASC stock number N6316), *bio2.1* (NASC stock number N6329) and *suc5* mutant lines (SAIL_367_D07, *suc5.4*; SALK_092412, *suc5.5*) were obtained from the Nottingham Arabidopsis Stock Centre (NASC). *Agrobacterium tumefaciens* GV3101 (Holsters *et al*., 1980) was used for Arabidopsis transformation by floral dip (Clough and Bent, 1998). *Escherichia coli* strain DH5α (Hanahan, 1983) was used for all cloning steps.

### RNA isolation and cDNA synthesis

RNA was isolated from 7 DAF siliques of wild-type and *suc5* mutants using the ‘seeds and siliques’ protocol described by Oñate-Sánchez and Vicente-Carbajosa (2008). cDNA was synthesized from 500 ng total RNA using high-capacity RNA-to-cDNA Master Mix (Applied Biosystems, www.appliedbiosystems.com). *SUC5* and *ACTIN2* mRNA levels were determined by PCR on 1 μl cDNA using primers 1, 2 and 3 and *ACTIN2*-specific primers (Table S1).

### Subcellular localization of SUC5

For SUC5 fusion proteins, the SUC5 coding sequence was amplified using primers SUC5+1f-BspHI and SUC5+2079r-BspHI (Table S1), the stop codon was removed, and flanking *Bsp*HI sites were introduced. The sequence was inserted into pSS87 (Schneider *et al*., 2011), producing GFP–SUC5, or into pCS120 (Dotzauer *et al*., 2010), yielding SUC5–GFP. Protoplast generation and transformation were performed as described by Drechsel *et al*. (2011) and Abel and Theologis (1994). Particle bombardment was performed as described by Klepek *et al*. (2005).

### Characterization of *bio1*, *bio2* and *suc5* single mutants, and generation of homozygous *bio suc5* double mutants

The position of the T-DNA insertion in the *suc5.4* mutant was determined by sequencing PCR fragments amplified using primers LB2 and AtSUC5g540f (Table S1). The position of the double insertion in *suc5.5* was determined using primers LBa1 and AtSUC5g540f or LBa1 and AtSUC5g2136r (Table S1). Homozygous *bio1.1* and *bio2.1* plants were obtained from heterozygous, biotin-watered *BIO1*/*bio1.1* or *BIO2*/*bio2.1* plants as described by Schneider *et al*. (1989) and Patton *et al*. (1998), and crossed with homozygous *suc5.4* or *suc5.5* plants. Resulting seeds (cross 0 seeds) were germinated on soil, and the presence of the *suc5.4* or *suc5.5* insertion was determined by PCR. Seeds from plants carrying *suc5.4* or *suc5.5* alleles (cross 1 seeds) were germinated on biotin-free medium. Pale seedlings (indicating homozygosity for *bio1.1* or *bio2.1*) were rescued on high-biotin medium, transferred to soil, and watered with biotin. Eventually, cross 2 seeds were germinated either on biotin-free medium (to re-confirm that 100% of the seedlings turned pale) or on high-biotin medium, and homozygosity for *suc5.4* or *suc5.5* was determined by PCR.

### Uptake measurements of biotin and sucrose in embryos

Siliques (8 DAF) from wild-type and *suc5.4* mutant plants were collected and dissected using fine forceps. Developing seeds were selected, and zygotic embryos at the upturned U stage were transferred into 25 mm sodium phosphate buffer, pH 7.0. For every uptake experiment, 50 embryos were incubated at 22°C in 200 μl solution containing 25 mm NaHPO_4_ (pH 5.5) and 2 mm CaCl_2_ plus the radiolabelled substrate. Indicated concentrations of ^14^C-biotin or ^14^C-sucrose were added. The incubation time was 6 h for biotin and 90 min for sucrose. Low biotin concentrations (10–50 μm) were chosen because ^14^C-biotin is in short supply. After incubation, samples were filtered on glass microfibre 696 filters (VWR, www.vwr.com), and washed 5 times for 5 min using an excess of distilled H_2_O to remove unincorporated radioactivity. Incorporated radioactivity was determined by scintillation counting. Incubation with each substrate and at each concentration was performed three times using independently isolated embryos.

### Generation of p*SUC5*/reporter lines

For construction of p*SUC5*/*sGFP*, 2030 bp of p*SUC5* were amplified using primers AtSUC5-2030f and AtSUC5-1r (Table S1), and introduced into pEP/pUC19 (Imlau *et al*., 1999) via *Hin*dIII and *Not*I sites. From the resulting vector, pEP-S5-GFP, p*SUC5*/*sGFP* was excised using *Hin*dIII and *Sac*I, and cloned into the respective sites of pAF16 (Stadler 2005b). This construct was used for Arabidopsis transformation.

For construction of p*SUC5*/*tmGFP9*, a genomic 1152 bp fragment encoding the 232 N-terminal amino acids of *STP9* (Schneidereit *et al*., 2003) was excised from plasmid pMH4 (Stadler *et al*., 2005b) using *Nco*I and inserted into the unique *Nco*I site separating p*SUC5* and the GFP open reading frame in pEP-S5-GFP. From the resulting plasmid, the 3916 bp p*SUC5*/*tmGFP9* cassette was excised using *Hin*dIII/*Sac*I, and cloned into the respective sites of pAF16, yielding pMH21, which was used for Arabidopsis transformation.

### Confocal microscopy

For detection of GFP fluorescence, images were produced using made a confocal microscope (Leica TCS SPII, www.leica-microsystems.com) as described previously (Stadler *et al*., 2005b). The excitation wavelength for GFP was 488 nm. Confocal images were processed using Leica confocal software 2.

### Analyses of TAG and fatty acids

Fatty acid methyl esters of pooled Arabidopsis seeds were obtained and identified essentially as described by Hoffmann *et al*., 2008.

## Notes

Figure S1 Young seedlings (a) and fully developed rosettes (b) of plants grown with supplementation of various amounts of biotin.

Figure S2 Seedlings (a) and fully developed rosettes (b) of the same plants germinated on biotin-containing (1mm) agar medium for 10 days, transferred to soils and supplemented with various amounts of biotin.

Table S1 Primer sequences.

## References

[b1] Abel S, Theologis A (1994). Transient transformation of Arabidopsis leaf protoplasts: a versatile experimental system to study gene expression. Plant J.

[b2] Baldet P, Ruffet ML (1996). Biotin synthesis in higher plants: isolation of a cDNA encoding *Arabidopsis thaliana bioB*-gene product equivalent by functional complementation of a biotin auxotroph mutant *bioB105* of *Escherichia coli* K12. C. R. Acad. Sci. III.

[b3] Baud S, Boutin J-P, Miquel M, Lepiniec L, Rochat C (2002). An integrated overview of seed development in *Arabidopsis thaliana* ecotype WS. Plant Physiol. Biochem.

[b4] Baud S, Wuillème S, Lemoine R, Kronenberger J, Caboche M, Lepiniec L, Rochat C (2005). The sucrose transporter AtSUC5 specifically expressed in the endosperm is involved in early seed development in Arabidopsis. Plant J.

[b5] Baud S, Mendoza MS, To A, Harscoet E, Lepiniec L, Debreucq B (2007). WRINKLED1 specifies the regulatory action of LEAFY COTYLEDON2 towards fatty acid metabolism during seed maturation in Arabidopsis. Plant J.

[b6] Caspari T, Stadler R, Sauer N, Tanner W (1994). Structure/function relationship of the *Chlorella* glucose/H^+^ symporter. J. Biol. Chem.

[b7] Cernac A, Andre C, Hoffmann-Benning S, Benning C (2006). WRI1 is required for seed germination and seedling establishment. Plant Physiol.

[b9] Chen LQ, Qu XQ, Hou BH, Sosso D, Osorio S, Fernie AR, Frommer WB (2012). Sucrose efflux mediated by SWEET proteins as a key step for phloem transport. Science.

[b10] Clough SJ, Bent AF (1998). Floral dip: a simplified method for *Agrobacterium*-mediated transformation of *Arabidopsis thaliana*. Plant J.

[b11] Ding G, Che P, Ilarslan H, Wurtele ES, Nikolau BJ (2012). Genetic dissection of methylcrotonyl CoA carboxylase indicates a complex role for mitochondrial leucine catabolism during seed development and germination. Plant J.

[b12] Dotzauer D, Wolfenstetter S, Eibert D, Schneider S, Dietrich P, Sauer N (2010). Novel PSI domains in plant and animal H^+^-inositol symporters. Traffic.

[b13] Drechsel G, Bergler J, Wippel K, Sauer N, Vogelmann K, Hoth S (2011). C-terminal armadillo repeats are essential and sufficient for association of the plant U-box armadillo E3 ubiquitin ligase SAUL1 with the plasma membrane. J. Exp. Bot.

[b14] Focks N, Benning C (1998). *wrinkled1*: a novel, low-seed-oil mutant of Arabidopsis with a deficiency in the seed-specific regulation of carbohydrate metabolism. Plant Physiol.

[b16] Hanahan D (1983). Studies on transformation of *Escherichia coli* with plasmids. J. Mol. Biol.

[b17] Hoffmann M, Wagner M, Abbadi A, Fulda M, Feussner I (2008). Metabolic engineering of ω3-VLCPUFA production by an exclusively acyl-CoA-dependent pathway. J. Biol. Chem.

[b18] Holsters M, Silva B, Van Vliet F (1980). The functional organization of the nopaline *A. tumefaciens* plasmid pTiC58. Plasmid.

[b19] Imlau A, Truernit E, Sauer N (1999). Cell-to-cell and long-distance trafficking of the green fluorescent protein in the phloem and symplastic unloading of the protein into sink tissues. Plant Cell.

[b20] Klepek Y-S, Geiger D, Stadler R, Klebl F, Landouar-Arsivaud L, Lemoine R, Hedrich R, Sauer N (2005). Arabidopsis POLYOL TRANSPORTER5, a new member of the monosaccharide transporter-like superfamily, mediates H^+^-symport of numerous substrates, including *myo-*inositol, glycerol, and ribose. Plant Cell.

[b21] Knowles JR (1989). The mechanism of biotin-dependent enzymes. Annu. Rev. Biochem.

[b22] Ludwig A, Stolz J, Sauer N (2000). Plant sucrose–H^+^ symporters mediate the transport of vitamin H. Plant J.

[b23] Matsuraka C, Saitoh T, Hirose T, Oshugi R, Perata P, Yamaguchi J (2000). Sugar uptake and transport in rice embryo. Expression of companion cell-specific sucrose transporter (OsSUT1) induced by sugar and light. Plant Physiol.

[b24] Meyer S, Lauterbach C, Niedermeier M, Barth I, Sjolund R, Sauer N (2004). Wounding enhances expression of AtSUC3, a sucrose transporter from Arabidopsis sieve elements and sink tissues. Plant Physiol.

[b25] Muralla R, Chen E, Sweeney C, Gray JA, Dickerman A, Nikolau BJ, Meinke DW (2008). A bifunctional locus (*BIO1–BIO3*) required for biotin biosynthesis in Arabidopsis. Plant Physiol.

[b26] Nikolau BJ, Ohlrogge JB, Wurtele ES (2003). Plant biotin-containing carboxylases. Arch. Biochem. Biophys.

[b27] Ohlrogge JB, Jaworski JG (1997). Regulation of fatty acid synthesis. Annu. Rev. Plant Physiol. Plant Mol. Biol.

[b28] Oñate-Sánchez L, Vicente-Carbajosa J (2008). DNA-free RNA isolation protocols for *Arabidopsis thaliana*, including seeds and siliques. BMC Res. Notes.

[b29] Patton DA, Johnson M, Ward ER (1996). Biotin synthase from *Arabidopsis thaliana*. cDNA isolation and characterization of gene expression. Plant Physiol.

[b30] Patton D, Schetter AL, Franzmann LH, Nelson K, Ward ER, Meinke DW (1998). An embryo-defective mutant of Arabidopsis disrupted in the final step of biotin synthesis. Plant Physiol.

[b31] Penfield S, Rylott EL, Gilday AD, Graham S, Larson TR, Graham IA (2004). Reserve mobilization in the Arabidopsis endosperm fuels hypocotyl elongation in the dark, is independent of abscisic acid, and requires *PHOSPHOENOLPYRUVATE CARBOXYKINASE1*. Plant Cell.

[b32] Prasad PD, Wang H, Kekuda R, Fujita T, Fey Y-J, Devoe LD, Leibach FH, Ganapathy V (1998). Cloning and functional expression of a cDNA encoding mammalian sodium-dependent vitamin transporter mediating the uptake of pantothenate, biotin, and lipoate. J. Biol. Chem.

[b33] Reinders A, Sivitz AB, Ward J (2012). Evolution of plant sucrose uptake transporters. Front. Plant Sci.

[b34] Sauer N (2007). Molecular physiology of higher plant sucrose transporters. FEBS Lett.

[b35] Schneider T, Dinkins R, Robinson K, Shellhammer J, Meinke DW (1989). An embryo-lethal mutant of *Arabidopsis thaliana* is a biotin auxotroph. Dev. Biol.

[b36] Schneider S, Hulpke S, Schulz A (2011). Vacuoles release sucrose via tonoplast-localised SUC4-type transporters. Plant Biol.

[b37] Schneidereit A, Scholz-Starke J, Büttner M (2003). Functional characterization and expression analyses of the glucose-specific AtSTP9 monosaccharide transporter in pollen of Arabidopsis. Plant Physiol.

[b38] Sherson SM, Hemmann G, Wallace G, Forbes S, Germain V, Stadler R, Bechtold N, Sauer N, Smith SM (2000). Monosaccharide/proton symporter AtSTP1 plays a major role in uptake and response of Arabidopsis seeds and seedlings to sugars. Plant J.

[b39] Sivitz AB, Reinders A, Johnson ME, Krentz AD, Grof CP, Perroux JM, Ward JM (2007). Arabidopsis sucrose transporter AtSUC9. High-affinity transport activity, intragenic control of expression, and early flowering mutant phenotype. Plant Physiol.

[b40] Smith AG, Croft MT, Moulin M, Webb ME (2007). Plants need their vitamins too. Curr. Opin. Plant Biol.

[b42] Stadler R, Lauterbach C, Sauer N (2005a). Cell-to-cell movement of GFP reveals post-phloem transport in the outer integument and identifies symplastic domains in Arabidopsis seeds and embryos. Plant Physiol.

[b43] Stadler R, Wright KM, Lauterbach C, Amon G, Gahrtz M, Feuerstein A, Oparka KJ, Sauer N (2005b). Expression of GFP-fusions in Arabidopsis companion cells reveals non-specific protein trafficking into sieve elements and identifies a novel post-phloem domain in roots. Plant J.

[b44] Stolz J, Meier S, Sauer N, Schweizer E (1999). Identification of the plasma membrane H^+^–biotin symporter of *Saccharomyces cerevisiae* by rescue of a fatty acid-auxotrophic mutant. J. Biol. Chem.

[b47] Weaver LM, Yu FU, Wurtele ES, Nikolau BJ (1996). Characterization of the cDNA and gene coding for the biotin synthase of *Arabidopsis thaliana*. Plant Physiol.

[b48] Will A, Grassl R, Erdmenger J, Caspari T, Tanner W (1998). Alteration of substrate affinities and specifities of *Chlorella* hexose/H+ symporters by mutations and constructions of chimeras. J. Biol. Chem.

[b49] Wolfenstetter S, Wirsching P, Dotzauer D, Schneider S, Sauer N (2012). Routes to the tonoplast: the sorting of tonoplast transporters in Arabidopsis mesophyll protoplasts. Plant Cell.

