# Supplementary material for: SUCROSE TRANSPORTER 5 supplies Arabidopsis embryos with biotin and affects triacylglycerol accumulation
Source: Plant J. 2012 Dec 31;73(3):392–404. doi: 10.1111/tpj.12037 (PMC3787789; doi:10.1111/tpj.12037)
Supplement: Figure S1 — Young seedlings (a) and fully developed rosettes (b) of plants grown with supplementation of various amounts of biotin. [file tpj0073-0392-sd1.docx]

**SUPPORTING INFORMATION (Pommerrenig *et al.*)**


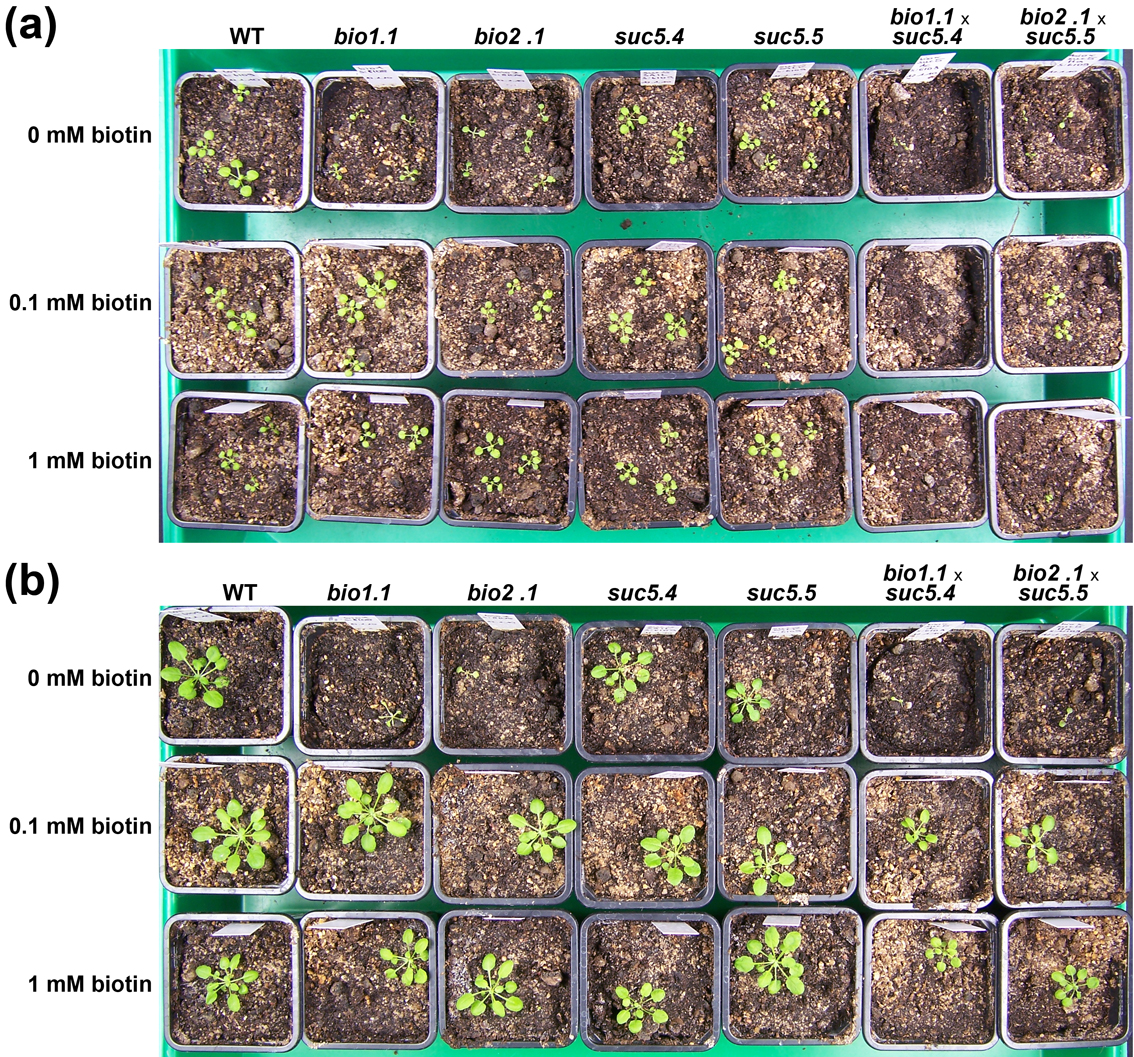


**Figure S1.** Young seedlings and fully developed rosettes of plants grown with different supplements of biotin.

Seeds of wt and mutant plants were sown directly on potting soil and watered with the indicated amount of biotin.

(a) After 24 d in the growth chamber (21°C, 8 h light, 16 h dark), double mutant seedlings were hardly visible, for the reasons explained in Figures 5 and 6 of the manuscript. After this picture had been taken, all but the largest seedlings were removed.

(b) Same plants [14 d older than in (a)] that carried a *bio1.1* or *bio2.1* mutant allele and that were not supplemented with biotin (0 mM biotin) did not develop rosette leaves. Pot size = 6 x 6 cm.
