# Supplementary material for: SUCROSE TRANSPORTER 5 supplies Arabidopsis embryos with biotin and affects triacylglycerol accumulation
Source: Plant J. 2012 Dec 31;73(3):392–404. doi: 10.1111/tpj.12037 (PMC3787789; doi:10.1111/tpj.12037)
Supplement: Figure S2 — Seedlings (a) and fully developed rosettes (b) of the same plants germinated on biotin-containing (1mm) agar medium for 10 days, transferred to soils and supplemented with various amounts of biotin. [file tpj0073-0392-sd2.docx]

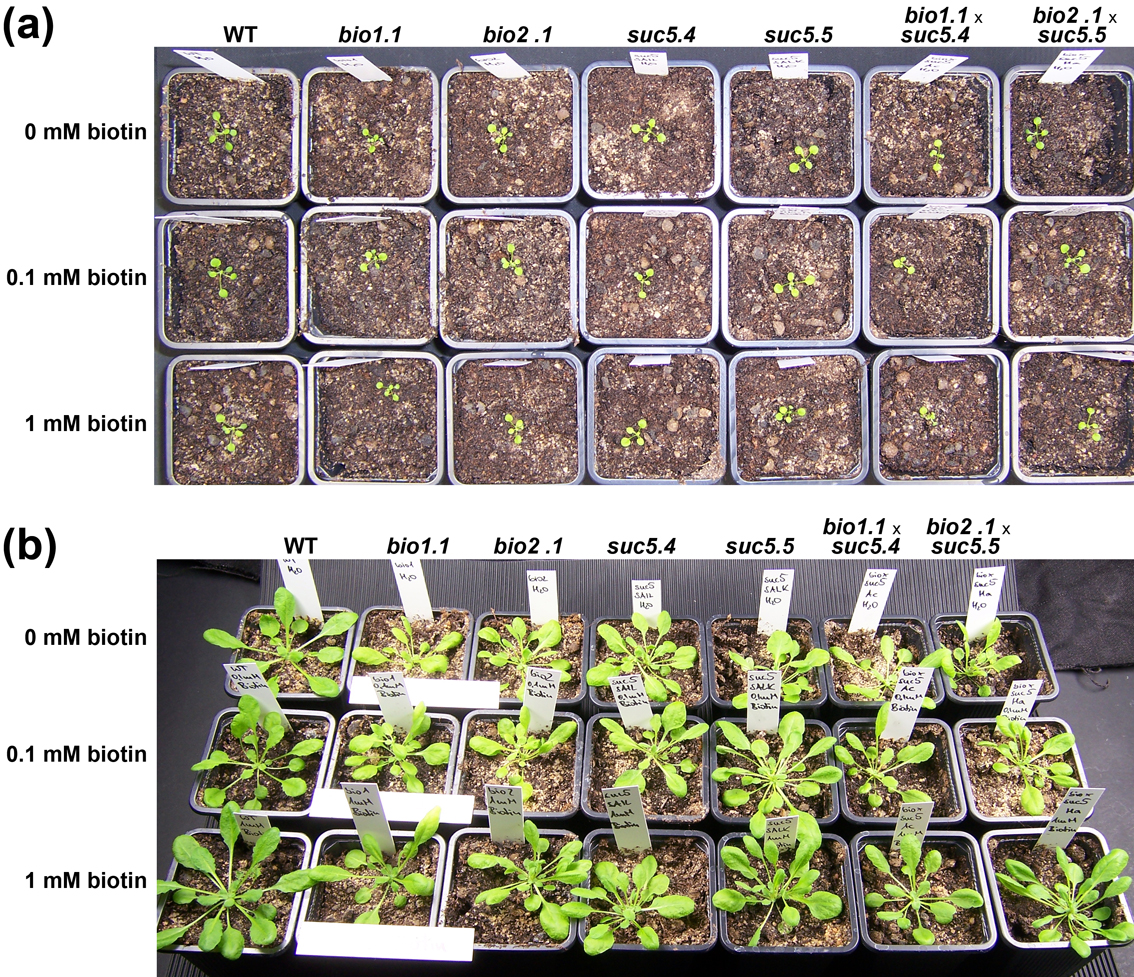


**Figure S2.** Seedlings and fully developed rosettes of the very same plants grown with different supplements of biotin.

(a) wt and mutant plants were first grown for 12 d on MS-medium supplemented with 1 mM biotin and transferred to soil (21°C, 8h light, 16 h dark) when all seedlings had the same size.

(b) Rosettes [30 d older than in (a)] were formed in plants with a *bio1.1* or *bio2.1* mutant allele even when these plants were not supplemented with biotin (0 mM biotin). Pot size = 6 x 6 cm.
