# Supplementary material for: SUCROSE TRANSPORTER 5 supplies Arabidopsis embryos with biotin and affects triacylglycerol accumulation
Source: Plant J. 2012 Dec 31;73(3):392–404. doi: 10.1111/tpj.12037 (PMC3787789; doi:10.1111/tpj.12037)
Supplement: Table S1 — Primer sequences. [file tpj0073-0392-sd3.docx]

| Primer name | Purpose | Primer sequence |
| --- | --- | --- |
|  |  |  |
| Act2g+846f | *ACT2* 5’ primer for RT-PCR | 5’-ATTCAGATGCCCAGAAGTCTTGTT-3’ |
| Act2g+1295r | *ACT2* 3’ primer for RT-PCR | 5’-GAAACATTTTCTGTGAACGATTCCT-3’ |
| AtSUC5-2030f | 5’ primer for pSUC5 with HindIII site | 5’-AAGCTTAACAATTTATGTAGTTTAGAACG-3’ |
| AtSUC5-1r | 3’ primer for pSUC5 with NcoI site | 5’-CCATGGTGAAAAGAAAAACGAGCAGACAA-3’ |
| AtSUC5g540f  (= primer 1 in Figure 2) | *SUC5* 5’ primer for *suc5.4* and *suc5.5* alleles (5’-end of insertion) and 5’ primer for RT-PCR (=primer 1 in Figure 2) | 5’-CGCAAACGCGTGTTTCTCCT-3’ |
| AtSUC5g1199r  (= primer 2 in Figure 2) | SUC5 3’ primer for RT-PCR (=primer 2 in Figure 2) | 5’-TCCGGCTTTAATACCACTGC-3’ |
| AtSUC5g2136r  (= primer 3 in Figure 2) | SUC5 3’ primer for *suc5.5* allele (3’-end of insertion) and 3’ primer for RT-PCR (=primer 3 in Figure 2) | 5’-tgcacaacaatactgtattagatgg-3’ |
| SUC5+1f-BspHI | SUC5 5’ primer for generating SUC5-GFP and GFP-SUC5 fusions | 5’-TCATGAGAGCCTTGGAAGCAGAAAG-3’ |
| SUC5+2079r-BspHI | SUC5 3’ primer for generating SUC5-GFP and GFP-SUC5 fusions | 5’-GGGCTATGGGATTCCATGTCATGA-3’ |
| LB2 | T-DNA left border of SAIL-lines | 5’-GCTTCCTATTATATCTTCCCAAATTACCAATACA-3’ |
| LBa1 | T-DNA left border of SALK-lines | 5’-TGGTTCACGTAGTGGGCCATCG-3’ |

**Table S1.** Primer sequences
